# Supplementary material for: Dimensional reduction by geometrical frustration in a cubic antiferromagnet composed of tetrahedral clusters
Source: Nat Commun. 2021 Jul 19;12:4382. doi: 10.1038/s41467-021-24636-1 (PMC8289872; doi:10.1038/s41467-021-24636-1)
Supplement: Supplementary file 2 — Supplementary Information [file 41467_2021_24636_MOESM2_ESM.docx]

**Supplementary Information for “Dimensional reduction by geometrical frustration in a cubic antiferromagnet composed of tetrahedral clusters”**

Ryutaro Okuma^1,2*@^, Maiko Kofu^3^, Shinichiro Asai^1^, Maxim Avdeev^4,5^, Akihiro Koda^6^, Hirotaka Okabe^6^, Masatoshi Hiraishi^6^, Soshi Takeshita^6^, Kenji M. Kojima^6**^, Ryosuke Kadono^6^, Takatsugu Masuda^1^, Kenji Nakajima^3^, and Zenji Hiroi^1^

Institute for Solid State Physics, University of Tokyo, Chiba, 277-8581, Japan. ^2^Okinawa Institute of Science and Technology Graduate University, Okinawa, 904-0495, Japan. ^3^Materials and Life Science Division, J-PARC Center, Japan Atomic Energy Agency, Tokai, Ibaraki, 319-1195, Japan. ^4^Australian Nuclear Science and Technology Organization, New Illawarra Road, Lucas Heights, NSW 2234, Australia. ^5^School of Chemistry, The University of Sydney, NSW 2006, Australia. ^6^Institute of Materials Structure Science, High Energy Accelerator Research Organization (KEK-IMSS), Tsukuba, Ibaraki, 305-0801, Japan.

*Present address: Clarendon Laboratory, University of Oxford, Oxford, OX1 3PU, UK. **Present address: Center for Molecular and Materials Science, TRIUMF, Vancouver, V6T 2A3 Canada.

^@^e-mail: ryutaro.okuma@gmail.com

1. CRYSTAL StructurE refinementS of pharmacosiderite

A nuclear structural refinement of a deuterated pharmacosiderite, (D_3_O)Fe_4_(OD)_4_(AsO_4_)_3_•5.5D_2_O, was performed for powder neutron diffraction data using the Fullprof Suite^1^. Because the sample contained hexagonal D_2_O ice as a minor phase, a two-phase fit was performed, as shown in Supplementary Fig. 1a, which yielded the structural parameters listed in Supplementary Table 1. The initial structure models employed were based on the parameters deduced from the previous powder X-ray diffraction (XRD) analysis^2^ for pharmacosiderite and the hexagonal structure of water ice *I*_h_ for D_2_O ice^3^. According to ref. 16, Fe1, As1, O1, and O2 are fully occupied, forming a framework with cavities where O3 and O4 statistically reside forming water molecules or hydronium ions. Thanks to the large neutron cross section of deuterium, deuterium positions in these units were also determined, which was difficult to achieve in the previous XRD study. D2 of the hydroxy group (Supplementary Fig. 1b) is fully ordered and located at a 4*e* site, similar to O2. Concerning the deuterium atoms related to water, namely, D1 and D3, they inevitably possess orientational disorders because of the low point group symmetry of a water molecule. D1 and D3 are placed at 12*i* positions with fractional occupancies around the O3 and O4 atoms, respectively. The occupancy of D1 and O3 is fixed at half such that the water molecule made from both sites must take one of two orientations around the face-centre position of the unit cell. The trigonal pyramidal unit made of O4 and D3 is compatible with a hydronium ion (D_3_O^+^) or a disordered water molecule. The fractional occupancies of O4 and D3 were determined to fulfil the chemical formula at 87.5 %. A soft constraint over the bond length between the D and O atoms was applied to all deuterium atoms. The fitting appears reasonably good in Supplementary Fig. 1a, but the agreement factor *R*_wp_ in the final refinement remains as high as 26.5 %. This is probably due to incomplete deuteration and large occupational disorder at the O3, O4, D1, and D3 sites.

Two kinds of crystal water or hydronium molecules in the framework are highlighted in Supplementary Figs. 1c and 1d, and their influence on magnetic interactions was considered. Water molecules made of D1 and O3 must have little effect on magnetic interactions because they are distant from Fe and not involved in the magnetic paths. In contrast, water molecules or hydronium ions made of D3 and O4 must affect the magnetic path more seriously because they form a hydrogen bond with D2, which is a part of the hydroxide ligand of Fe. Thus, the orientational and occupational disorder of the D3–O4 unit may moderate the intracluster interaction to some extent but with little influence on the intercluster interaction.


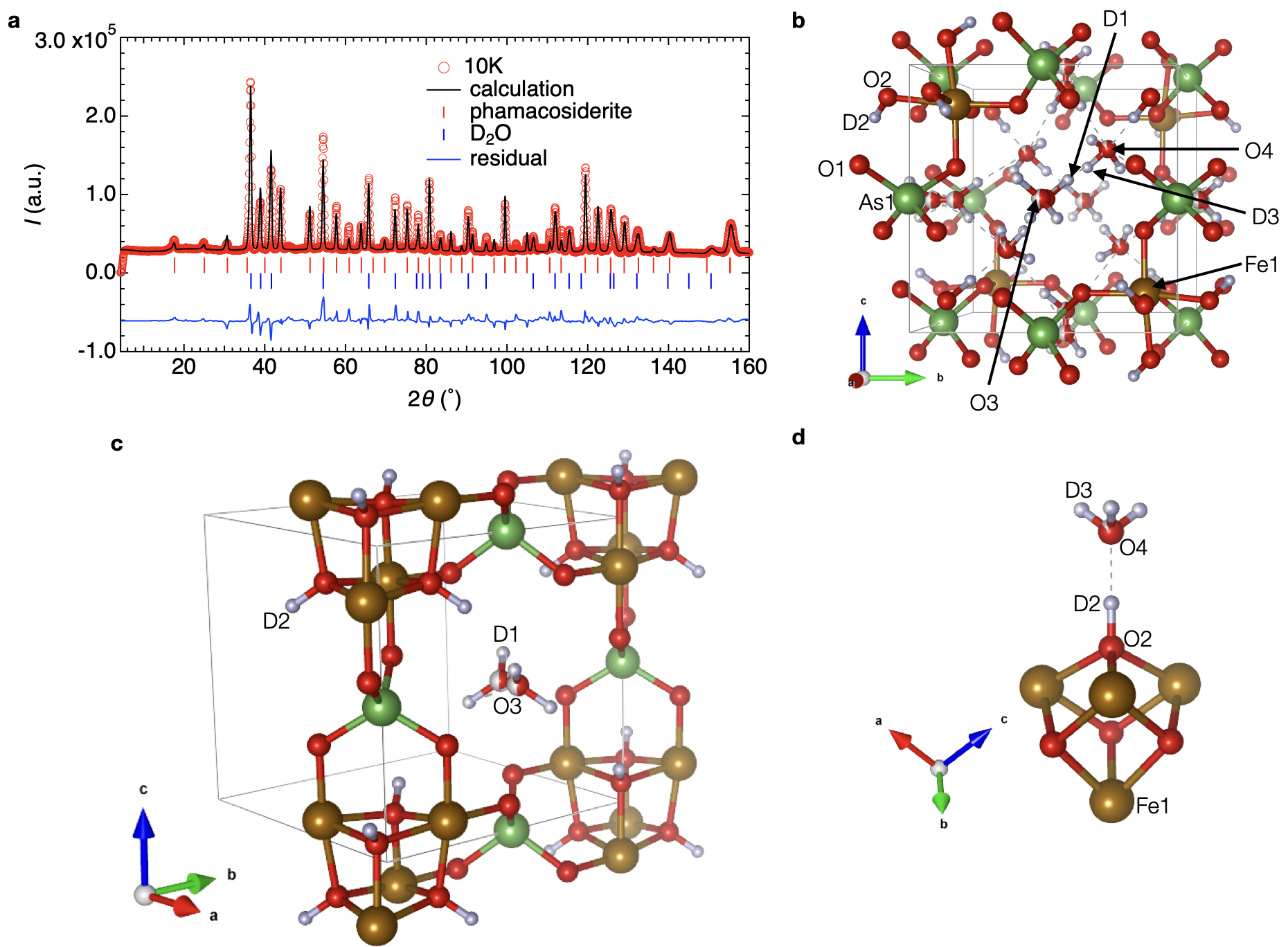


**Supplementary Fig. 1 | Nuclear structural refinement on the powder neutron diffraction data.** **a.** Red marks, the black line, and blue line represent raw data at 10K, a fit, and a residual, respectively. The red and blue bars indicate the positions of the Bragg peaks of pharmacosiderite and ice, respectively. **b**. Refined crystal structure of pharmacosiderite. **c**. Disorder of a water molecule near the face centred position in pharmacosiderite. **c**. Disorder of a water molecule or hydronium ion near a hydroxy group in pharmacosiderite.

| Formula | | | | (D_3_O)Fe_4_(OD)_4_(AsO_4_)_3_•5.5D_2_O | | |
| --- | --- | --- | --- | --- | --- | --- |
| Space group | | | | *P*-43*m* | | |
| *a* / Å | | | | 7.99822(8) | | |
| *V* / Å^3^ | | | | 511.659(9) | | |
| *Z* | | | | 1 | | |
| Atom | Wyckoff position | *x* | *y* | *z* | *B*_iso_ | Occupancy |
| Fe | 4*e* | 0.13972(63) | 0.13972(63) | 0.13972(63) | 1 | 1 |
| As | 3*d* | 0.5 | 0 | 0 | 1 | 1 |
| O1 | 12*i* | 0.14129(68) | 0.14129(68) | 0.37936(85) | 1 | 1 |
| O2 | 4*e* | 0.88733(53) | 0.88733(53) | 0.88733(53) | 1 | 1 |
| O3 | 6*g* | 0.5 | 0.04543(156) | 0.5 | 1 | 0.5 |
| O4 | 4*e* | 0.70465(73) | 0.70465(73) | 0.70465(73) | 1 | 0.875 |
| D1 | 12*i* | 0.57478(102) | 0.11877(183) | 0.42527(102) | 3 | 0.5 |
| D2 | 4*e* | 0.81455(51) | 0.81455(51) | 0.81455(51) | 3 | 1 |
| D3 | 12*i* | 0.62774(69) | 0.62774(69) | 0.73458(109) | 3 | 0.667 |

| Fomula | | | D_2_O | | |
| --- | --- | --- | --- | --- | --- |
| Space group | | | *P*6_3_/*mmc* | | |
| *a* / Å | | | 4.50072(9) | | |
| *c* / Å | | | 7.33031(24) | | |
| *V* / Å^3^ | | | 128.593(6) | | |
| *Z* | | | 1 | | |
| Atom | *x* | *y* | *z* | *B*_iso_ | Occ. |
| O | 1/3 | 2/3 | 0.05986(81) | 1 | 1 |
| D1 | 0.45797(68) | 0.91595(136) | 0.02377(69) | 3 | 1/2 |
| D2 | 1/3 | 2/3 | 0.18954(114) | 3 | 1/2 |

**Supplementary Table 1 | Structural parameters of pharmacosiderite and D_2_O *I*_h_ ice obtained via the Rietveld refinements.**

1. Analysis of mössbauer spectra

The low-temperature Mössbauer spectra of pharmacosiderite were analysed based on the Blume–Tjon model^4^. It is assumed in the model that a local magnetic field from spins takes either +*h* or –*h* with probabilities per unit time *W*_1_ and *W*_2_, respectively, and flips between the two states randomly. In a magnetically ordered state, *W* = (*W*_1_ + *W*_2_)/2 and α = (*W*_1_ – *W*_2_)/(*W*_1_ + *W*_2_) correspond to the average frequency of magnetic fluctuation and the degree of magnetic ordering, respectively^5^. The angle made by the principal axis of the electric field gradient (three-fold axis in pharmacosiderite) and the local field is designated as *θ*. The results of fitting are good, as shown in Fig. 2b. The refined parameters are presented in Supplementary Table 2. The values of the isomer shift (IS) and quadrupole splitting (QS) determined by fitting the paramagnetic data at 6 K were used for the analyses of the 4 and 2.8 K data. The peak width of the underlying Lorentzian function *Γ* was refined for each dataset considering variable inhomogeneity. The data at 4 K were analysed as paramagnetic because the refined α value was negligible.

| *T* (K) | *Γ* (mm/sec) | IS (mm/sec) | QS (mm/sec) | *h* (T) | *W* (MHz) | *α* (%) | *θ* (°) |
| --- | --- | --- | --- | --- | --- | --- | --- |
| 6 | 0.441(5) | 0.494(2) | 0.1176(6) | 0 | – | – | – |
| 4 | 1.42(9) | 0.494 | 0.1176 | 61 | 254(23) | 0 | 49(2) |
| 2.8 | 0.86(3) | 0.494 | 0.1176 | 61(5) | 313(72) | 57(5) | 45(1) |

**Supplementary Table 2 | Mössbauer parameters of pharmacosiderite. Fixed parameters in the fitting are given without uncertainty.**

The obtained average local frequency of magnetic fluctuation is approximately 300 MHz, which is the same order as that detected by μSR in Section 3. The direction of the local field is 45(1)° at 2.8 K, which is roughly consistent with the magnetic structure determined by neutron diffraction in Section 4: *θ* takes cos^-1^(2/√6) ~ 35° in the Γ_5_ structure (Supplementary Fig. 2).


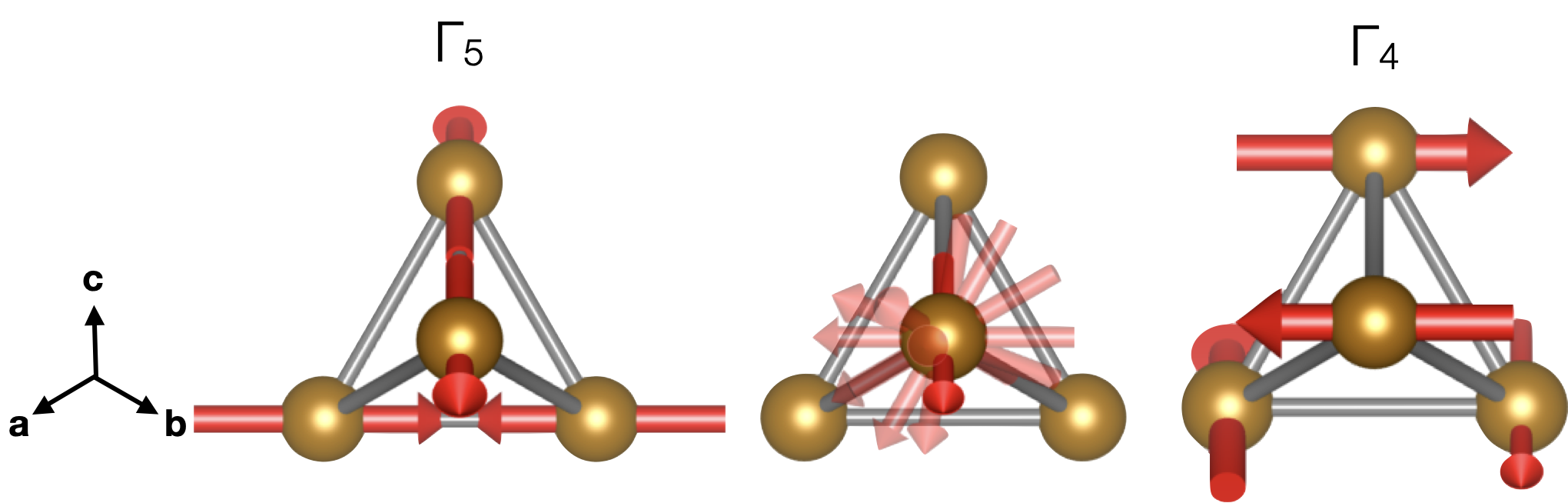


**Supplementary Fig. 2 | Local spin arrangement viewed along the [111] direction, which is the principal axis of the electric field gradient at an Fe site.** In the Γ_5_ magnetic structure two of the four spins are perpendicular to the viewing direction, and the other 2 spins make an angle of ~35º to the viewing direction.

1. LOCAL SPIN DYNAMICS PROBED BY µSR

Supplementary Fig. 3 shows the time evolution of the positron decay asymmetry [*A*(*t*), which we call the time spectrum] in the paramagnetic phase of K-pharmacosiderite [KFe_4_(AsO_4_)_3_(OH)_4_·nH_2_O]. Here, we used a pharmacosiderite with a K ion inside the cage instead of a hydronium ion; the magnetic properties were found to be insensitive to the type of cage ion. In general, *A*(*t*) is proportional to the instantaneous muon polarisation projected along the *z* axis parallel to the initial muon beam direction; *A*(*t*) = *A*_0_*P_z_*(*t*), where *A*_0_ is the instrumental asymmetry and is equal to ~0.23. The time spectrum at 59 K well above *T*_N_ under a zero external field (ZF) is characterised by a slow exponential damping that overlaps with a sinusoidal oscillation. This oscillatory signal are attributed to neither magnetic order nor muonium formation, the former of which is excluded by the fact that *A*(0) exhibits full polarisation. The origin of the oscillation may be attributed to the formation of a local atomic cluster consisting of a muon and a small number (≤ 2) of nearby atoms having nuclear magnetic moments. One such classical example is the F^–^–μ^+^–F^–^ (FμF^–^) complex observed in alkali metal fluorides, where two farad nuclei (spin *I* = 1/2) and one muon form a local three-spin system that gives rise to coherent spin precession^6^; the FμF^–^ state is a muonic analogue of hydrogen difluoride (FHF^−^), which is known as a prototype of strong hydrogen bonding^7^. Another example is found in sodium alanate, NaAlH_4_, where the alanate ion [AlH_4_]^–^ substitutes the role of fluorine in the interstitial muon to form an HμH-bonding state^8^. These examples indicate a common tendency for a muon to behave as a pseudo-hydrogen and form a hydrogen bond with negatively charged light ions. Thus, we presume that a part of the implanted muons in pharmacosiderite also form a similar local spin complex with the H of the hydroxyl bases, giving the observed sinusoidal oscillation.

Assuming a static collinear geometry with μ^+^ at the centre of the line joining the two other nuclear spins (*I* = 1/2), the time evolution of muon polarisation as a cubic average is calculated by solving a simple three-spin model to yield

$$\begin{aligned} \boldsymbol{G}_{\mathbf{3S}}\left( \boldsymbol{t} \right)\boldsymbol{=}\frac{\boldsymbol{1}}{\boldsymbol{6}}\left[ \boldsymbol{3+}\mathbf{cos}\left( \sqrt{\boldsymbol{3}}\boldsymbol{\omega}_{\mathbf{d}}\boldsymbol{t} \right)\boldsymbol{+}\boldsymbol{\alpha}_{\boldsymbol{+}}\mathbf{cos}\left( \boldsymbol{\beta}_{\boldsymbol{+}}\boldsymbol{\omega}_{\mathbf{d}}\boldsymbol{t} \right)\boldsymbol{+}\boldsymbol{\alpha}_{\boldsymbol{-}}\mathbf{cos}\left( \boldsymbol{\beta}_{\boldsymbol{-}}\boldsymbol{\omega}_{\mathbf{d}}\boldsymbol{t} \right) \right]\boldsymbol{\#(}\boldsymbol{SEQ Equation \backslash* ARABIC}\mathbf{1}\boldsymbol{)} \end{aligned}\boldsymbol{,}$$

where *α*_±_ = 1± 1/√3, *β*_±_ = (3 3√± )/2, and *ω*_d_ is the dipolar interaction frequency.

$$\begin{aligned} \boldsymbol{\omega}_{\mathbf{d}}\boldsymbol{=2}\boldsymbol{\gamma}_{\boldsymbol{\mu}}\boldsymbol{\gamma}_{\boldsymbol{I}}\boldsymbol{/}\boldsymbol{r}^{\boldsymbol{3}}\boldsymbol{\#(}\boldsymbol{SEQ Equation \backslash* ARABIC}\mathbf{2}\boldsymbol{),} \end{aligned}$$

where *γ_I_* and *r* are the gyromagnetic ratios of nuclear spin (*γ_I_*/2π = 42.58 MHz/T for ^1^H) and the distance between μ^+^ and the nucleus. On the other hand, it is also known that a muon may occupy a site bonding with one of hydroxyl bases^9, 10^, leading to a two-spin model given by the following function:

$$\begin{aligned} \boldsymbol{G}_{\mathbf{2S}}\left( \boldsymbol{t} \right)\boldsymbol{=}\frac{\boldsymbol{1}}{\boldsymbol{6}}\left[ \boldsymbol{1+}\mathbf{cos}\left( \boldsymbol{\omega}_{\mathbf{d}}\boldsymbol{t} \right)\boldsymbol{+2}\mathbf{cos}\left( {\frac{\boldsymbol{1}}{\boldsymbol{2}}\boldsymbol{\omega}}_{\mathbf{d}}\boldsymbol{t} \right)\boldsymbol{+2}\mathbf{cos}\left( \frac{\boldsymbol{3}}{\boldsymbol{2}}\boldsymbol{\omega}_{\mathbf{d}}\boldsymbol{t} \right) \right]\boldsymbol{\#} \end{aligned}\boldsymbol{(}\boldsymbol{SEQ Equation \backslash* ARABIC}\mathbf{3}\boldsymbol{).}$$

The time spectrum under a longitudinal field of 0.05 T exhibits exponential damping, whereas the spin depolarisation due to the nuclear dipolar field is quenched in the high field limit (*B*_0_ >> *ω*_d_/*γ*_μ_ ∼10^−3^ T). This indicates that a considerable fraction of implanted muons are subjected to exponential depolarisation due to fluctuation of local fields exerted by paramagnetic moments.


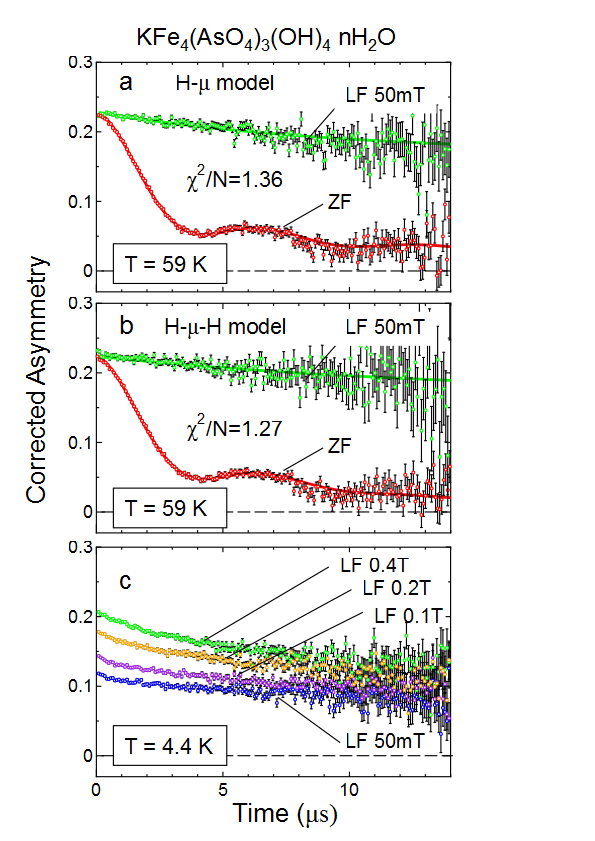


**Supplementary Fig. 3 | a, b. Typical µSR time spectra observed in K-pharmacosiderite at 59 K under ZF and longitudinal fields (LF) of 0.05 T.** A clear sinusoidal oscillation is seen under ZF, which is readily quenched upon applying a small LF of 0.05 T. The relaxing signal is analysed by assuming an H–µ bonding (**a**) and an H–µ–H bonding (**b**) (see text). Besides, the remaining exponential damping under LF is attributed to the fluctuating Fe moments. **c**, Spectra observed at 4.4 K below *T*_N_ showing a recovery of *A*(0) with increasing longitudinal fields, which indicates that the fast depolarization exceeding the time resolution of muon pulses of ~0.1 µs is induced by a quasi-static internal field.

The time spectra below *T*_N_, shown in Supplementary Fig. 3c, exhibits a recovery of *A*(0) with increasing longitudinal fields, indicating that a part of the signal undergoes a fast depolarisation induced by a quasi-static local field; a similar feature is observed for H_3_O-pharmacosiderite, as shown in Fig. 3c in the main text. The time evolution for this component under LF = *B*_0_ (*x* = *B*_0_/*B*_loc_) is approximately given by

$$\begin{aligned} \boldsymbol{G}_{\boldsymbol{z}}^{\mathbf{stat}}\left( \boldsymbol{t,x} \right)\boldsymbol{\simeq}\boldsymbol{G}_{\boldsymbol{\infty}}\left( \boldsymbol{x} \right)\boldsymbol{+}\left[ \boldsymbol{1}\boldsymbol{-}\boldsymbol{G}_{\boldsymbol{\infty}}\left( \boldsymbol{x} \right) \right]\mathbf{exp}\left( \boldsymbol{-}\boldsymbol{\sigma}_{\mathbf{s}}^{\boldsymbol{2}}\boldsymbol{t}^{\boldsymbol{2}} \right)\boldsymbol{\#(}\boldsymbol{SEQ Equation \backslash* ARABIC}\mathbf{4}\boldsymbol{),} \end{aligned}$$

$$\begin{aligned} \boldsymbol{G}_{\boldsymbol{\infty}}\left( \boldsymbol{x} \right)\boldsymbol{=}\frac{\boldsymbol{3}\boldsymbol{x}^{\boldsymbol{2}}\boldsymbol{-}\boldsymbol{1}}{\boldsymbol{4}\boldsymbol{x}^{\boldsymbol{2}}}\boldsymbol{+}\frac{\boldsymbol{(}\boldsymbol{x}^{\boldsymbol{2}}\boldsymbol{-}\boldsymbol{1}\boldsymbol{)}^{\boldsymbol{2}}}{\boldsymbol{16}\boldsymbol{x}^{\boldsymbol{3}}}\mathbf{log}\left[ \frac{\boldsymbol{(x+1}\boldsymbol{)}^{\boldsymbol{2}}}{\boldsymbol{(x}\boldsymbol{-}\boldsymbol{1}\boldsymbol{)}^{\boldsymbol{2}}} \right]\boldsymbol{\#(}\boldsymbol{SEQ Equation \backslash* ARABIC}\mathbf{5}\boldsymbol{),} \end{aligned}$$

where *G*_∞_(*x*) corresponds to the powder average of the probability that the net local field **B**_loc_ + **B**_0_ is parallel to the initial muon spin polarisation, and *σ*_s_ (> 1 μs^−1^) denotes the relaxation rate. In addition, a remaining component exhibits an exponential depolarisation under LF, which may be described by

$$\begin{aligned} \boldsymbol{G}_{\boldsymbol{z}}^{\mathbf{dyn}}\left( \boldsymbol{t,}\boldsymbol{\omega}_{\boldsymbol{\mu}} \right)\boldsymbol{\simeq}\mathbf{exp}\left[ \boldsymbol{-}\boldsymbol{\Lambda}_{\boldsymbol{d}}\left( \boldsymbol{\omega}_{\boldsymbol{\mu}} \right)\boldsymbol{t} \right]\boldsymbol{\#(}\boldsymbol{SEQ Equation \backslash* ARABIC}\mathbf{6}\boldsymbol{),} \end{aligned}$$

$\begin{aligned} \boldsymbol{\Lambda}_{\mathbf{d}}\left( \boldsymbol{\omega}_{\boldsymbol{\mu}} \right)\boldsymbol{\simeq}\frac{\boldsymbol{2}\boldsymbol{\delta}_{\boldsymbol{\mu}}^{\boldsymbol{2}}\boldsymbol{\nu}}{\boldsymbol{\omega}_{\boldsymbol{\mu}}^{\boldsymbol{2}}\boldsymbol{+}\boldsymbol{\nu}^{\boldsymbol{2}}}\boldsymbol{\#(}\boldsymbol{SEQ Equation \backslash* ARABIC}\mathbf{7}\boldsymbol{),} \end{aligned}$

where *ω*_μ_ = *γ*_μ_*B*_0_, *δ*_μ_ is the muon–Fe hyperfine field, and *ν* is the fluctuation rate of *δ*_μ_. Notably, Supplementary Eq. (7) becomes least dependent on *B*_0_ for *ν* ≫ *ω*_μ_, which mimics the behaviour of the exponentially depolarizing component for *A*(*t*).

Considering these three components, the time spectra in Supplementary Fig. 3 are analysed using a variety of models and are best reproduced by the following model relaxation functions:

$$\begin{aligned} \boldsymbol{A}\left( \boldsymbol{t} \right)\boldsymbol{=}\boldsymbol{A}_{\boldsymbol{0}}\left[ \left( \boldsymbol{1}\boldsymbol{-}\boldsymbol{f} \right)\boldsymbol{G}_{\mathbf{3S}}\left( \boldsymbol{t} \right)\boldsymbol{+f}\boldsymbol{G}_{\boldsymbol{z}}^{\mathbf{dyn}}\left( \boldsymbol{t,}\boldsymbol{\omega}_{\boldsymbol{\mu}} \right) \right]\left( \boldsymbol{T>}\boldsymbol{T}_{\mathbf{N}} \right)\boldsymbol{\#(}\boldsymbol{SEQ Equation \backslash* ARABIC}\mathbf{8}\boldsymbol{)} \end{aligned}\boldsymbol{,}$$

$$\begin{aligned} \boldsymbol{A}\left( \boldsymbol{t} \right)\boldsymbol{=}\boldsymbol{A}_{\boldsymbol{0}}\left[ \left( \boldsymbol{1}\boldsymbol{-}\boldsymbol{f} \right)\boldsymbol{G}_{\mathbf{3S}}\left( \boldsymbol{t} \right)\boldsymbol{+f}\boldsymbol{G}_{\boldsymbol{z}}^{\mathbf{dyn}}\left( \boldsymbol{t,}\boldsymbol{\omega}_{\boldsymbol{\mu}} \right) \right]\boldsymbol{G}_{\boldsymbol{z}}^{\mathbf{stat}}\left( \boldsymbol{t,x} \right)\left( \boldsymbol{T<}\boldsymbol{T}_{\mathbf{N}} \right)\boldsymbol{\#(}\boldsymbol{SEQ Equation \backslash* ARABIC}\mathbf{9}\boldsymbol{),} \end{aligned}$$

where *f* is the fractional yield of the component exhibiting persistent dynamical modulation. The parameters deduced from the fitting analysis are summarised in Supplementary Table 3. The fitting analysis using *G*_2S_(*t*) instead of *G*_3S_(*t*) gives a slightly larger value of *χ*^2^/*N*, as shown in Supplementary Figs. 3a and 3b. In addition, the use of $G_{3S}\left( t \right)G_{z}^{\mathrm{dyn}}\left( t,\omega_{\mu} \right)$ instead of $G_{3S}\left( t \right)$ does not improve the fitting. Thus, we use the model given by Supplementary Eqs. (8) and (9) in the following discussion. It is plausible that the observed two magnetic sectors, (1 - *f*) and *f*, come from different muon sites, which are referred to μ(1) and μ(2), respectively. We note that the parameter *f* is common in both analyses below and above *T*_N_.

|  | H-μ model (*G*_2S_(*t*)) | H-μ-H model (*G*_3S_(*t*)) |
| --- | --- | --- |
| *f* | 0.30 | 0.21 |
| *ω*_d_ / 2π [MHz] | 0.10189(8) | 0.06645(6) |
| *r* [nm] | 0.1554(4) | 0.1792(5) |
| *χ*^2^/*N* | 1.36 | 1.27 |

**Supplementary Table 3 | Summary of the fitting results assuming both cases of an H-μ bonding and an H-μ-H bonding.**

According to the above model, a muon at the μ(1) site forms an H–µ–H bonding state with nearby H atoms, and probes a quasi-static internal field **B**_loc_ from the ordered Fe moments below *T*_N_. The H–μ bond length is estimated to be 0.1792(5) nm from the magnitude of *ω_d_* (= 2π × 0.06645(6) MHz) deduced by the curve fit. On the other hand, a muon at the μ(2) site is exposed to an additional local field that possesses a persistent fluctuation, as indicated by the fact that a better fit is obtained by assuming a product of $G_{z}^{\mathrm{dyn}}(t,\omega_{\mu})$ and $G_{z}^{\mathrm{stat}}(t,x)$ below *T*_N_. Thus, the μ(2) probes two kinds of Fe moments with different dynamics: one is quasi-static and the other is fluctuating. This leads to the speculation that the magnetic order is somewhat inhomogeneous. The persistent fluctuating field at the μ(2) site for H_3_O-pharmacosiderite estimated by the analysis of the *B*_0_ dependence of *Λ*_d_ at 2 K using Supplementary Eq. (7) is *ν* ≃ 4.27(10) × 10^8^ s^−1^ with *δ*_μ_ ≃ 12.8(2) MHz, indicating a very small energy scale of 2 μeV, which is orders of magnitude smaller than that observed by the inelastic neutron scattering experiments. Thus, the μSR experiments detect much slower spin fluctuations in the LRO of the Fe moments, which must be different from the 2D spin fluctuations found by inelastic neutron scattering experiments. It is plausible to assume that the slow spin fluctuations come from the 1D defect generated in the decoupled 2D layers, as inferred by the SW calculations. The μ(2) site is located near the 1D defect and is subject to both quasi-static and fluctuating local fields. The latter is induced by a collective mode in the 1D defect, as shown in Fig. 5b in the main text, whereas the μ(1) site is located far from the 1D defect.

1. magnetic structure Analysis

The spin configuration of the **q** = 0 magnetic structure in pharmacosiderite was analysed based on the representation analysis using SARAh suite^11^. The Fe^3+^ atom resides at a 4*e* site with *x* ~ 0.14 in the space group of *P*–43*m*; 4 inequivalent sites in the unit cell are labelled as 1: (*x*, *x*, *x*), 2: (*x*, –*x*, –*x*), 3: (–*x*, *x*, – *x*), 4: (–*x*, –*x*, *x*)*.* The magnetic representation for a *q* = 0 structure can be decomposed into irreducible representations (IR) as follows:

Γ_q_ = Γ_2_ + 2Γ_2_ + 3Γ_4_ + 3Γ_5_^2^

Supplementary Table 4 lists twelve possible basis vectors (BV), $\psi_{1},\cdots,\psi_{12}$, with the components of (*m_a_*, *m_b_*, *m_c_*) for each of the four sites. Supplementary Fig. 4 illustrates all five types of spin configurations with zero net moment for the tetrahedron. Powder neutron diffraction intensities are calculated for these magnetic structures, as shown in Supplementary Fig. 5. Notably, there are intense (100) and (200) peaks in the experimental pattern of Fig. 3d, which are reproduced only for Γ_4_, ψ_4_ and Γ_5_, ψ_7_ – ψ_8_. Thus, we have carried out magnetic Rietveld refinements for the two magnetic structures, as shown in Supplementary Fig. 6. The agreement factor is significantly smaller for the Γ_5_ structure than for the Γ_4_ structure: *R*_wp_ = 29.8 % and 44.7 %, respectively. This fact indicates that the Γ_5_ structure is realized in pharmacosiderite. In addition, a small net moment along [001] is consistent with Γ_5_ structure, which is not allowed for Γ_4_^16^.

As shown in Supplementary Fig. 7, the magnetic order is long-range in nature, which is evident from the similar peak widths of the nuclear and magnetic (001) reflections. Estimation of the magnetic correlation length by microstructural analysis yielded a magnetic correlation length of 360.7(3) Å.

| IR | BV | Site | *m_a_* | *m_b_* | *m_c_* |
| --- | --- | --- | --- | --- | --- |
| Γ_2_ | ψ_1_ | 1 | 0.5774 | 0.5774 | 0.5774 |
|  |  | 2 | 0.5774 | -0.5774 | -0.5774 |
|  |  | 3 | -0.5774 | 0.5774 | -0.5774 |
|  |  | 4 | -0.5774 | -0.5774 | 0.5774 |
| Γ_3_ | ψ_2_ | 1 | 0.7071 | -0.7071 | 0 |
|  |  | 2 | 0.7071 | 0.7071 | 0 |
|  |  | 3 | -0.7071 | -0.7071 | 0 |
|  |  | 4 | -0.7071 | 0.7071 | 0 |
|  | ψ_3_ | 1 | 0.4082 | 0.4082 | -0.8165 |
|  |  | 2 | 0.4082 | -0.4082 | 0.8165 |
|  |  | 3 | -0.4082 | 0.4082 | 0.8165 |
|  |  | 4 | -0.4082 | -0.4082 | -0.8165 |
| Γ_4_ | ψ_4_ | 1 | 0 | -0.7071 | 0.7071 |
|  |  | 2 | 0 | 0.7071 | -0.7071 |
|  |  | 3 | 0 | 0.7071 | 0.7071 |
|  |  | 4 | 0 | -0.7071 | -0.7071 |
|  | ψ_5_ | 1 | 0.7071 | 0 | -0.7071 |
|  |  | 2 | -0.7071 | 0 | -0.7071 |
|  |  | 3 | -0.7071 | 0 | 0.7071 |
|  |  | 4 | 0.7071 | 0 | 0.7071 |
|  | ψ_6_ | 1 | -0.7071 | 0.7071 | 0 |
|  |  | 2 | 0.7071 | 0.7071 | 0 |
|  |  | 3 | -0.7071 | -0.7071 | 0 |
|  |  | 4 | 0.7071 | -0.7071 | 0 |
| Γ_5_ | ψ_7_ – ψ_8_ | 1 | 0 | 0.7071 | 0.7071 |
|  |  | 2 | 0 | -0.7071 | -0.7071 |
|  |  | 3 | 0 | -0.7071 | 0.7071 |
|  |  | 4 | 0 | 0.7071 | -0.7071 |
|  | ψ_7_ + 2ψ_8_ | 1 | 1 | 0 | 0 |
|  |  | 2 | 1 | 0 | 0 |
|  |  | 3 | 1 | 0 | 0 |
|  |  | 4 | 1 | 0 | 0 |
|  | ψ_9_ – ψ_10_ | 1 | 0.7071 | 0 | 0.7071 |
|  |  | 2 | -0.7071 | 0 | 0.7071 |
|  |  | 3 | -0.7071 | 0 | -0.7071 |
|  |  | 4 | 0.7071 | 0 | -0.7071 |
|  | ψ_9_ + 2ψ_10_ | 1 | 0 | 1 | 0 |
|  |  | 2 | 0 | 1 | 0 |
|  |  | 3 | 0 | 1 | 0 |
|  |  | 4 | 0 | 1 | 0 |
|  | ψ_11_ – ψ_12_ | 1 | 0.7071 | 0.7071 | 0 |
|  |  | 2 | -0.7071 | 0.7071 | 0 |
|  |  | 3 | 0.7071 | -0.7071 | 0 |
|  |  | 4 | -0.7071 | -0.7071 | 0 |
|  | ψ_11_ + 2ψ_12_ | 1 | 0 | 0 | 1 |
|  |  | 2 | 0 | 0 | 1 |
|  |  | 3 | 0 | 0 | 1 |
|  |  | 4 | 0 | 0 | 1 |

**Supplementary Table 4 | Irreducible representation (IR) and the components (*m_a_*, *m_b_*, *m_c_*) of basis vectors (BV) for the magnetic representation in the space group *P*−43*m* and the magnetic propagation vector q = 0.**


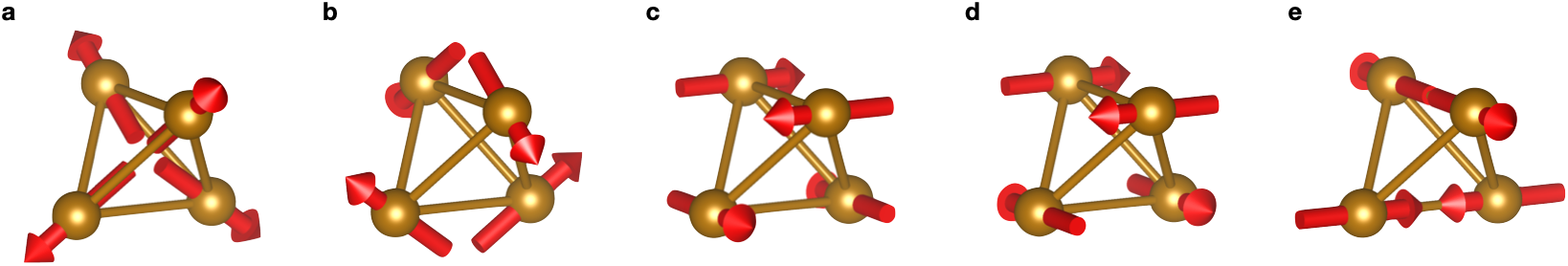
**Supplementary Fig. 4 | Representative basis vectors of irreducible representations for the q = 0 magnetic structure in pharmacosiderite. a.** Γ_2_, ψ_1_. **b.** Γ_3_, ψ_2_. **c.** Γ_3_, ψ_3_. **d.** Γ_4_, ψ_4_. **e.** Γ_5_, ψ_7_ – ψ_8_.

**

**Supplementary Fig. 5 | Simulated powder neutron diffraction intensities for the q = 0 magnetic structures shown in Supplementary Fig. 4.** The simulation for ψ_3_ is omitted since it is same as that of ψ_2_.


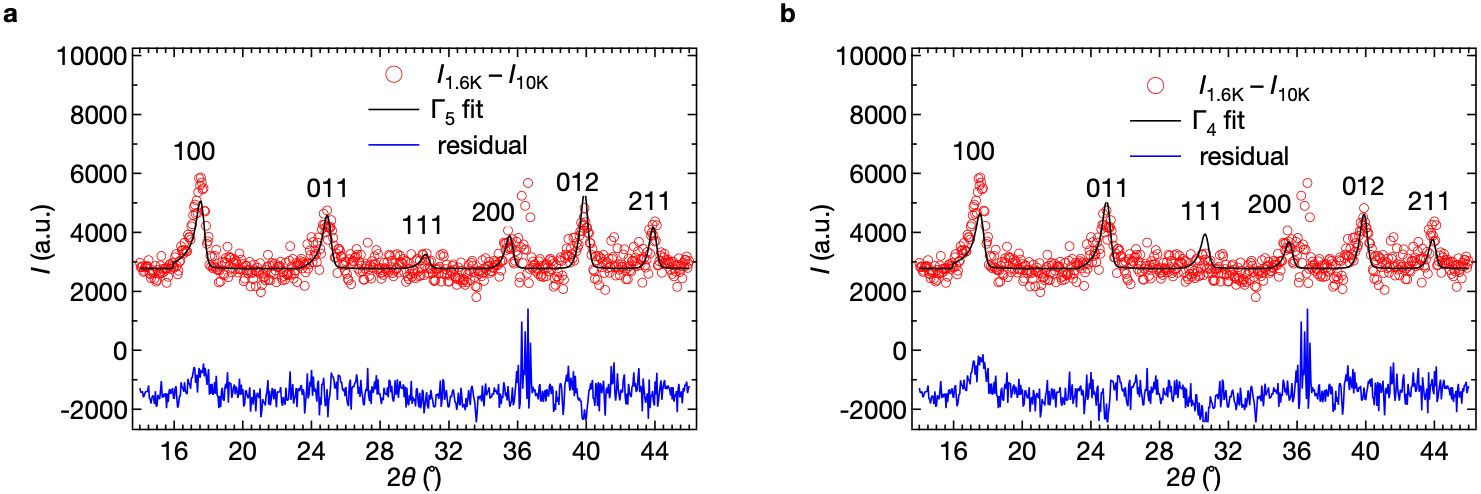


**Supplementary Fig. 6 | Powder neutron diffraction pattern of pharmacosiderite and its Rietveld fittings to a. Γ_4_ and ψ_4_ and b. Γ_5_ and ψ_7_ – ψ_8_ spin structures.** The red circles represent data taken at 1.6 K after the subtraction of the 10 K data as a reference of nuclear contributions. The black and blue lines represent a fit and a residual in each figure.

**Supplementary Fig. 7 | Comparison of (001) peak in the a. nuclear and b. magnetic diffraction.** The blue and red circles represent data taken at 10 K and data taken at 1.6 K after the subtraction of the 10 K data, respectively. The black lines represent fits to the Thompson–Cox–Hastings pseudo-Voigt function convoluted with the axial divergence asymmetry function installed in Fullprof^31^. FWHMs of the magnetic and nuclear peaks are 0.51 and 0.57 degrees, respectively.

1. Temperature dependence of the Dynamical structure factor

The temperature evolution of the powder-averaged dynamical structure factor *S*(*Q*, *E*) at *E*_i_ = 3.1 meV is shown in Supplementary Fig. 8. To estimate the energy dependence of the intensity, *S*(*Q*, *E*) is integrated along *Q* around the (001) reflection (Supplementary Fig. 9). The intense elastic component is located within ±0.1 meV (Supplementary Fig. 9a). An opening of a gap of ~1 meV is clearly observed upon cooling at the tail of the peak in Supplementary Fig. 9b. To estimate the magnetic diffraction pattern, *S*(*Q*, *E*) is integrated at an energy window between –0.1 and 0.1 meV (Supplementary Fig. 10a). The magnetic profile shown in the inset of Supplementary Fig. 10a resembles that of Fig. 3d in the elastic channel and is consistent with the *q* = 0, Γ_5_ structure. The temperature dependence of the (001) intensity is shown in Supplementary Fig. 10c, which is obtained by a Gaussian fit to each (001) peak shown in Supplementary Fig. 10b. The intensity increases below the Neél temperature *T*_N_ = 6 K and almost saturates below 2.5 K. Thus, the magnetic long-range order has already developed at 0.6 K with the ordered magnetic moment almost saturated at this temperature.

The temperature evolution of *S*(*Q*, *ω*) at *E*_i_ = 7.7 meV is shown in Supplementary Fig. 11. Dispersive modes indicative of spin-wave excitations are clearly observed below 7.2 K and disappear at 50 K. To estimate the energy dependence of the intensity, *S*(*Q*, *E*) is integrated along *Q* around the (001) reflection at 2 *Q* ranges of 0.7 Å^–1^ < *Q* < 0.86 Å^–1^ and 2.05 Å^–1^ < *Q* < 2.15 Å^–1^, where contributions from nuclear and magnetic Bragg reflections are minimal. The results are shown in Supplementary Fig. 12. For the former *Q* range, the 1.6 and 7.2 K data coincide with each other above 2 meV, whereas the 50 K data are larger than either, possibly owing to the contributions of phonons. For the latter *Q* range, the intensity is nearly temperature independent above 2 meV, indicative of the lack of magnetic contributions there. Thus, the magnetic contributions are mostly located below 2 meV. To estimate the total magnetic scattering, we used an intensity below 2 meV.


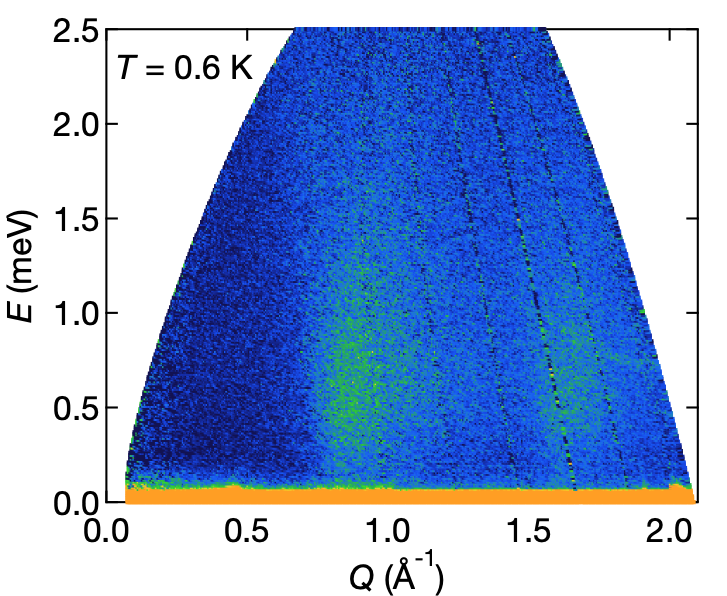

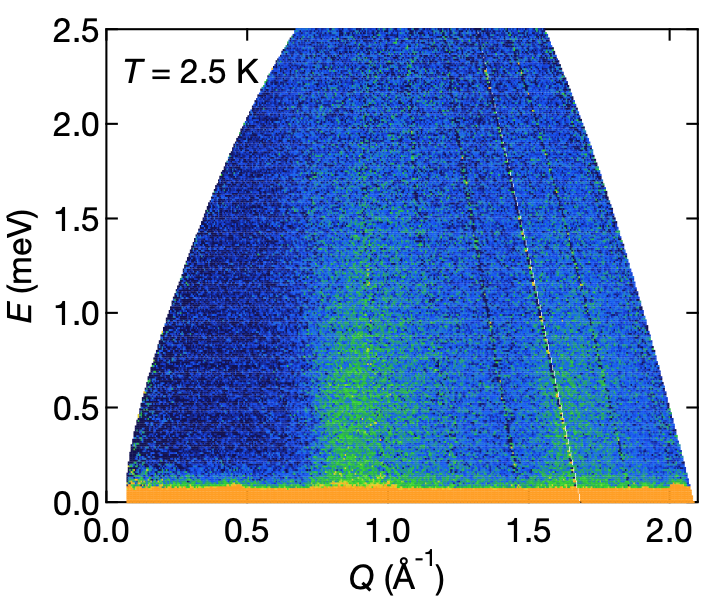

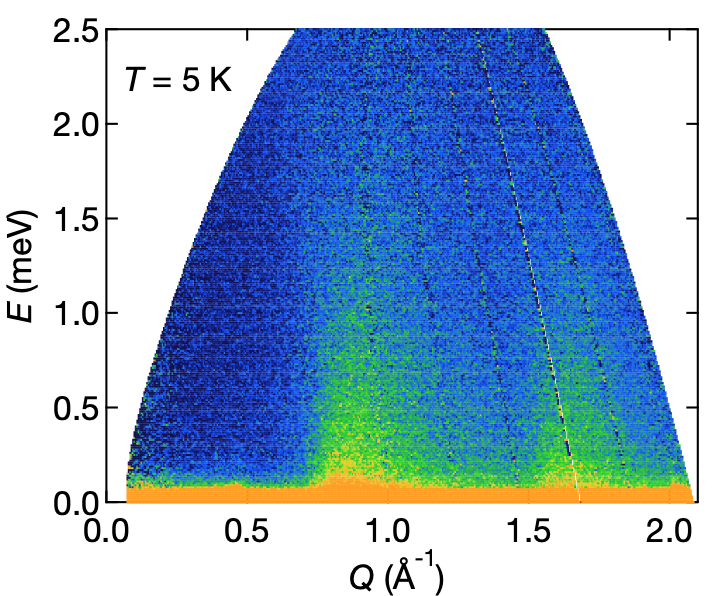

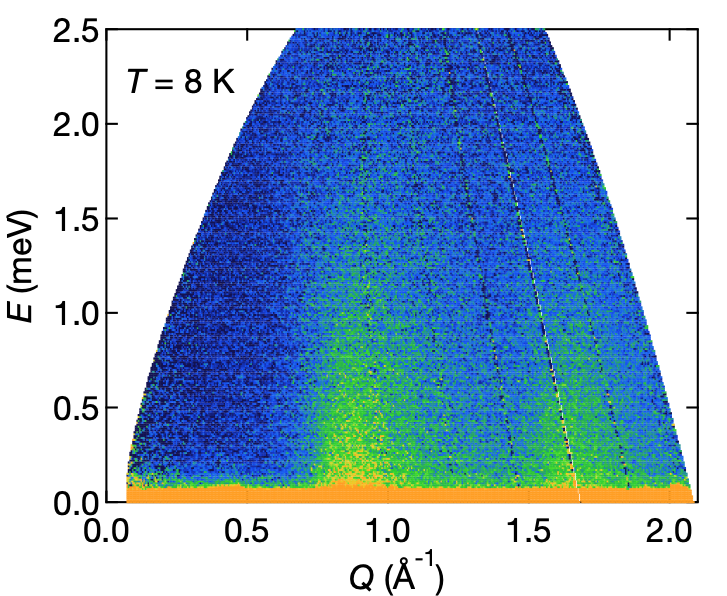

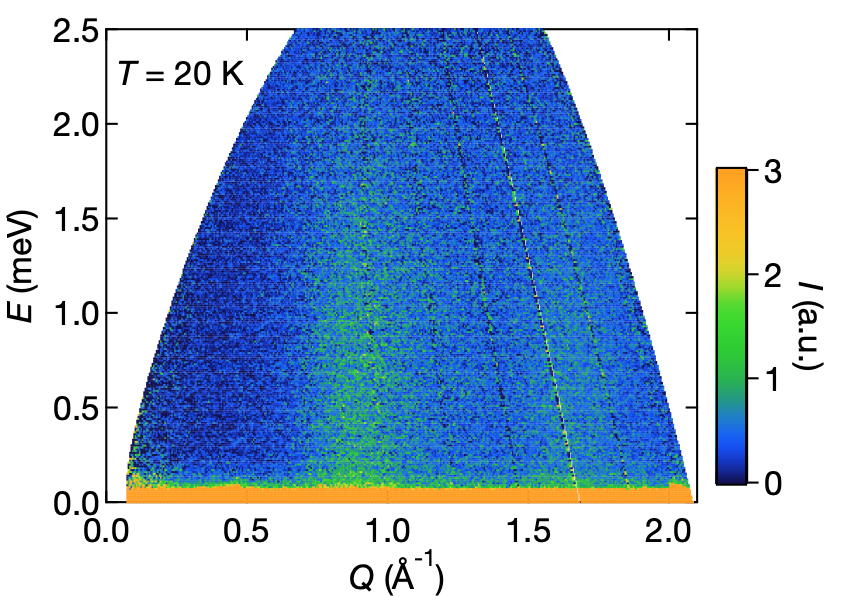


**Supplementary Fig. 8 | Powder-averaged dynamical structure factors *S*(*Q*, *E*) of pharmacosiderite at temperatures of 0.6, 2.5, 5, 8, and 20 K and at *E*_i_ = 3.1 meV.**


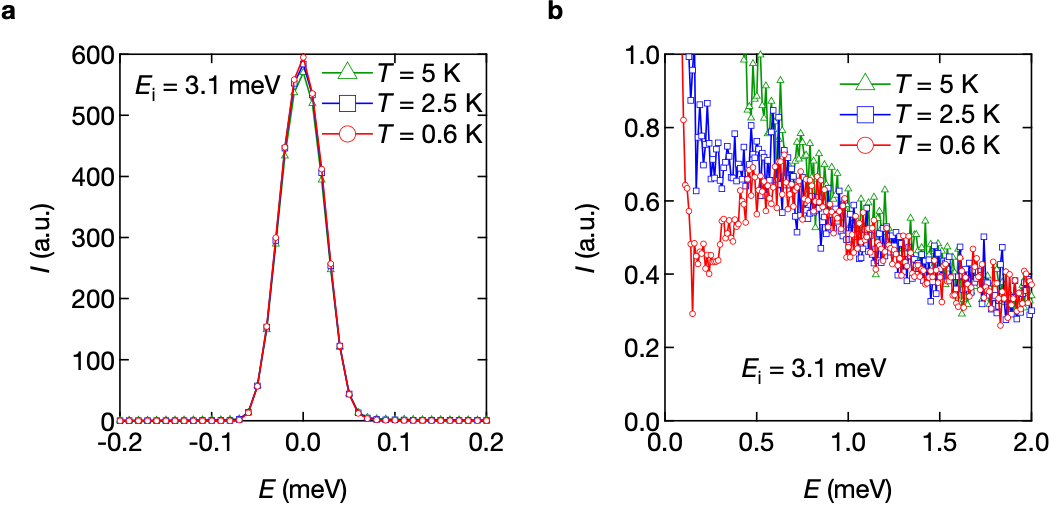


**Supplementary Fig. 9 | a. Neutron intensities obtained by integrating the *S*(*Q*, *E*)s along *Q* around the (001) reflection (0.7 Å^–1^ < *Q* < 0.86 Å^–1^) at temperatures of 0.6, 2.5, and 5 K and *E*_i_ = 3.1 meV.** The low-intensity regime is expanded in **b**.

**Supplementary Fig. 10 | Estimate of elastic contribution from the integrated dynamical structure factor. a.** Diffraction patterns estimated by integrating the *S*(*Q*, *E*) at an energy window between –0.1 and 0.1 meV at temperatures of 0.6 and 20 K. The inset shows a magnetic diffraction pattern obtained by subtracting the 20 K data from the 0.6 K data. **b.** Enlarged (001) reflection peaks at 0.6, 2.5, 5, 8, and 20 K with Gaussian fits represented by the solid lines. **c.** Temperature dependence of the intensity of the (001) peak estimated by the Gaussian fits in **b**.


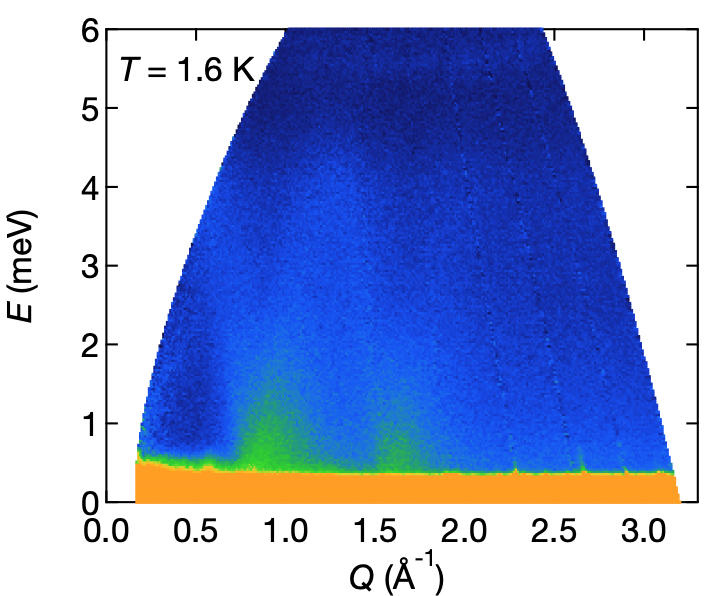

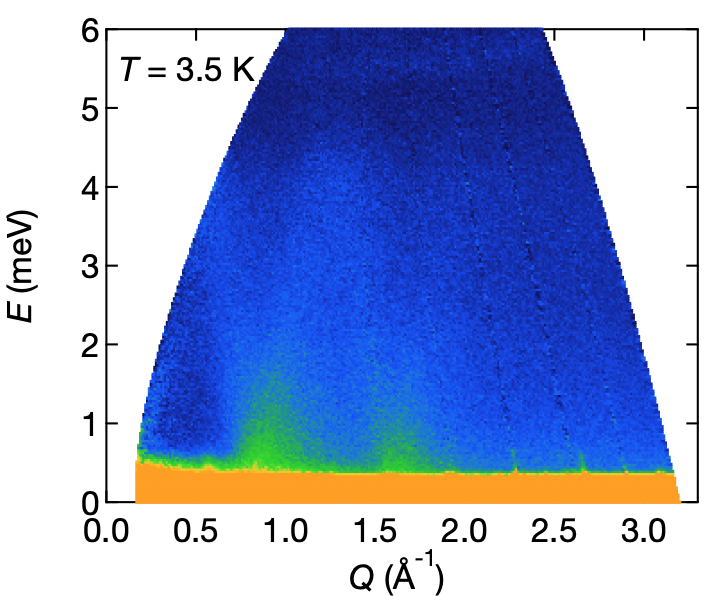

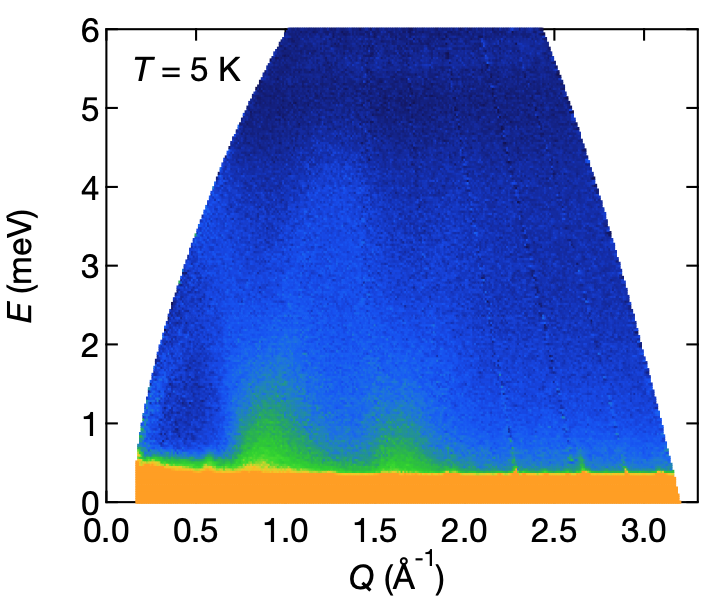

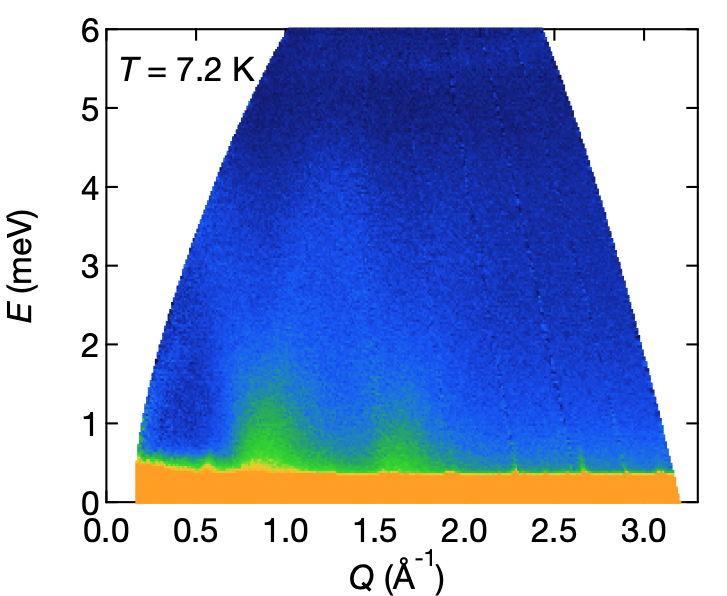

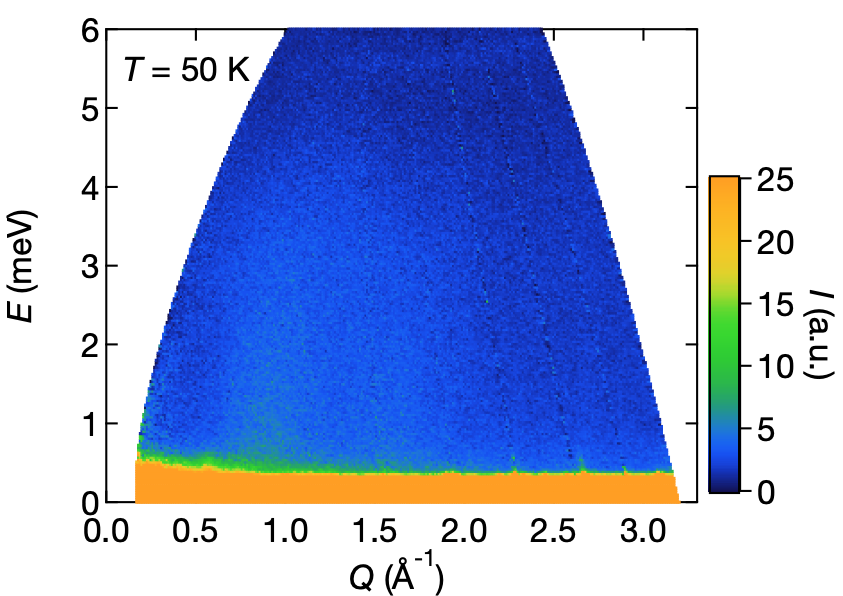


**Supplementary Fig. 11 | Powder-averaged dynamical structure factors *S*(*Q*, *E*) of pharmacosiderite at temperatures of 1.6, 3.5, 5, 7.2, and 50 K at *E*_i_ = 7.7 meV.**


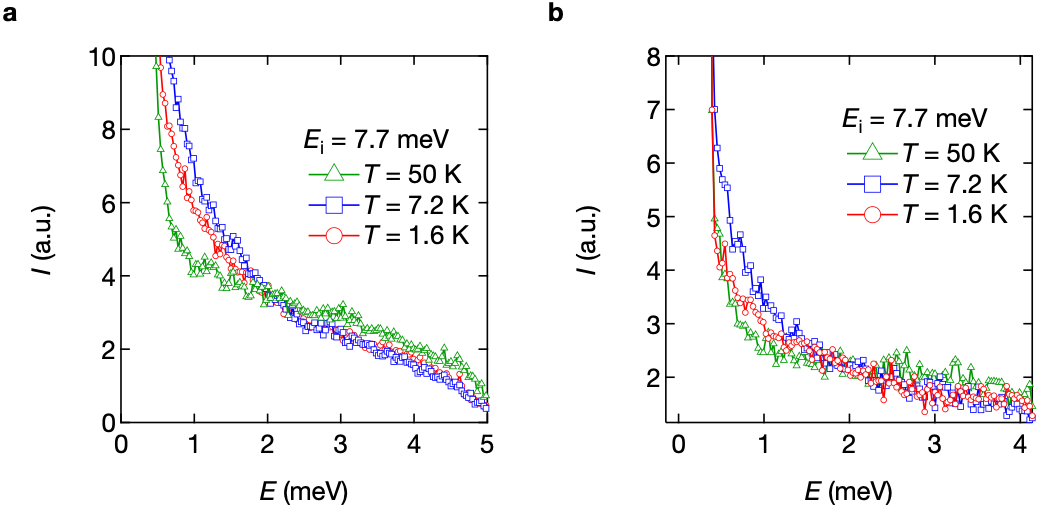


**Supplementary Fig. 12 | Estimate of magnetic contribution in the dynamical structure factor.** Neutron intensities obtained by integrating the *S*(*Q*, *E*)s along *Q* around the (001) reflection at **a.** 0.7 Å^–1^ < *Q* < 0.86 Å^–1^ and **b.** 2.05 Å^–1^ < *Q* < 2.15 Å^–1^, at both of which nuclear and magnetic Bragg contributions are minimal, at temperatures of 1.6, 7.2, and 50 K and *E*_i_ = 7.7 meV.

1. Comparison of the experiment to the linear spin wave theory

The energy and momentum dependences of the *S*(*Q*, *E*) of pharmacosiderite are analysed in terms of the linear spin wave theory (LSWT) using SpinW software^12^. The energy dependence of the spectra around the (001) reflection is shown in Supplementary Fig. 13a. The observed gap-like structure centred around 0.5 meV is qualitatively reproduced by the calculation, but the spectra are much sharper in the LSWT simulation. This broadening suggests that the actual magnon excitations are damped by strong fluctuations in the ordered state. In contrast, LSWT correctly captures the feature of momentum dependence in Fig. 13b, which results from two-dimensionality in the excitation, as revealed in the next section.


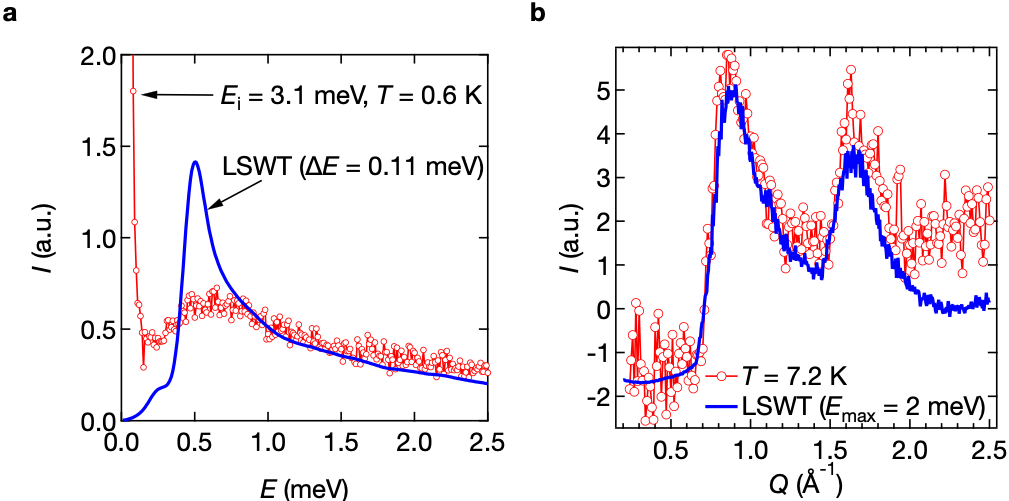


**Supplementary Fig. 13 | Analysis of the dynamical structure factor by LSWT for pharmacosiderite. a.** Energy dependence of the neutron intensity obtained by integrating the *S*(*Q*, *E*)s at *E*_i_ = 3.1 meV and *T* = 0.6 K over the *Q* range of 0.7 Å^–1^ < *Q* < 0.86 Å^–1^ around the (001) reflection. The solid blue line represents a simulated spectrum convoluted with the instrumental energy resolution of AMATERAS at *E*_i_ = 3.1 meV (*ΔE* = 0.11 meV). The simulated spectrum is normalized to the area intensity of the experimental data at 0.14 meV < *E* < 2.5 meV. **b**. Comparison of the momentum dependence of the *I* at 7.2 K shown in Fig. 4e in the main text with LSWT calculation. The blue line represents a calculated intensity obtained by integrating *S*(*Q*, *E*) at an energy range of 0 meV < *E* < 2 meV.

1. Analysis of THE equal time structure factor in terms of THE low dimensional scattering models

We consider paramagnetic scattering from classical spins in a crystal. The equal time structure factor or total scattering *I*(**Q**) at a three-dimensional wavevector **Q** is defined as the integral of the dynamical structure factor along all available energy. *I*(**Q**) can also be formulated as the scattering intensity from static objects as follows:

$\begin{aligned} \begin{aligned} \boldsymbol{I}\left( \mathbf{Q} \right)\boldsymbol{\propto}\int_{\boldsymbol{-\infty}}^{\boldsymbol{\infty}} \boldsymbol{d\omega}\boldsymbol{S}^{\boldsymbol{\mu\mu}}\left( \mathbf{Q}\boldsymbol{,\omega} \right)\boldsymbol{=}\sum_{\mathbf{r}\boldsymbol{,}\mathbf{r}\boldsymbol{'}} \boldsymbol{e}^{\boldsymbol{-}\boldsymbol{i}\left( \mathbf{Q}\boldsymbol{\cdot}\left( \mathbf{r}^{\boldsymbol{'}}\boldsymbol{-}\mathbf{r} \right) \right)}\overline{\boldsymbol{S}_{\mathbf{r}^{\boldsymbol{'}}}\left( \boldsymbol{0} \right)\boldsymbol{\cdot}\boldsymbol{S}_{\mathbf{r}}\left( \boldsymbol{0} \right)} \end{aligned}\boldsymbol{\#(}\boldsymbol{SEQ Equation \backslash* ARABIC}\mathbf{10}\boldsymbol{).} \end{aligned}$

Consider the powder-averaged total scattering from a *d*-dimensional short-range-ordered object. According to ref. 13, the scattering intensity is given by

$\begin{aligned} \boldsymbol{I}_{\boldsymbol{d}}\left( \boldsymbol{Q} \right)\boldsymbol{=}\sum_{\boldsymbol{\tau}} \boldsymbol{I}_{\boldsymbol{\tau}}\boldsymbol{\Phi}_{\boldsymbol{d}}\left( \boldsymbol{Q,\tau,D} \right)\boldsymbol{\#(}\boldsymbol{SEQ Equation \backslash* ARABIC}\mathbf{11}\boldsymbol{).} \end{aligned}$

Here, *Q*, *τ*, *D*, *I_τ_*, and *Φ_d_* represent the wavenumber, indices of the *d*-dimensional Bragg reflection, structure factor, and powder-averaged diffuse scattering from *τ* reflection, respectively. *D* is regarded as the width of the Bragg peaks and is proportional to the inverse of the correlation length of the short-range order. Specifically, *Φ_d_* in each dimension can be expressed in the following form:

$$\begin{aligned} \boldsymbol{\Phi}_{\boldsymbol{3}}\left( \boldsymbol{Q}_{\boldsymbol{0}}\boldsymbol{,\tau} \right)\boldsymbol{=}\frac{\boldsymbol{(2}\boldsymbol{\pi}\boldsymbol{)}^{\boldsymbol{3}}}{\boldsymbol{v}_{\boldsymbol{0}}\boldsymbol{\tau}^{\boldsymbol{2}}\sqrt{\boldsymbol{2}\boldsymbol{\pi}\boldsymbol{D}^{\boldsymbol{2}}}}\mathbf{exp}\left( \boldsymbol{-}\frac{\left( \boldsymbol{Q}_{\boldsymbol{0}}\boldsymbol{-}\boldsymbol{\tau} \right)^{\boldsymbol{2}}}{\boldsymbol{2}\boldsymbol{D}^{\boldsymbol{2}}} \right)\boldsymbol{\#(}\boldsymbol{SEQ Equation \backslash* ARABIC}\mathbf{12}\boldsymbol{),} \end{aligned}$$

$$\begin{aligned} \boldsymbol{\Phi}_{\boldsymbol{2}}\left( \boldsymbol{Q}_{\boldsymbol{0}}\boldsymbol{,\tau} \right)\boldsymbol{=}\frac{\boldsymbol{(2}\boldsymbol{\pi}\boldsymbol{)}^{\boldsymbol{2}}}{\boldsymbol{f}_{\boldsymbol{0}}\sqrt{\boldsymbol{2}\boldsymbol{\pi}\boldsymbol{D}^{\boldsymbol{2}}}}\int_{\boldsymbol{-\infty}}^{\boldsymbol{\infty}} \frac{\boldsymbol{dq}}{\boldsymbol{\tau}^{\boldsymbol{2}}\boldsymbol{+}\boldsymbol{q}^{\boldsymbol{2}}}\mathbf{exp}\left( \boldsymbol{-}\frac{\left( \boldsymbol{Q}_{\boldsymbol{0}}\boldsymbol{-}\sqrt{\boldsymbol{\tau}^{\boldsymbol{2}}\boldsymbol{+}\boldsymbol{q}^{\boldsymbol{2}}} \right)^{\boldsymbol{2}}}{\boldsymbol{2}\boldsymbol{D}^{\boldsymbol{2}}} \right)\boldsymbol{\#(}\boldsymbol{SEQ Equation \backslash* ARABIC}\mathbf{13}\boldsymbol{),} \end{aligned}$$

$$\begin{aligned} \boldsymbol{\Phi}_{\boldsymbol{1}}\left( \boldsymbol{Q}_{\boldsymbol{0}}\boldsymbol{,\tau} \right)\boldsymbol{=}\frac{\boldsymbol{(2}\boldsymbol{\pi}\boldsymbol{)}^{\boldsymbol{3}/\boldsymbol{2}}}{\boldsymbol{a}_{\boldsymbol{0}}\sqrt{\boldsymbol{2}\boldsymbol{\pi}\boldsymbol{D}^{\boldsymbol{2}}}}\boldsymbol{\#(}\boldsymbol{SEQ Equation \backslash* ARABIC}\mathbf{14}\boldsymbol{).} \end{aligned}$$

Here, *v*_0_, *f*_0_, and *a*_0_ represent the volume, surface, and length of each unit cell, respectively. To fit the *I*(*Q*) of pharmacosiderite by the low-dimensional order model, the background of thermal diffuse scattering from phonons is considered as follows:

$$\begin{aligned} \boldsymbol{I}_{\boldsymbol{d}}\left( \boldsymbol{Q} \right)\boldsymbol{=A+C}\boldsymbol{Q}^{\boldsymbol{2}}\boldsymbol{+}\sum_{\boldsymbol{\tau}} \boldsymbol{I}_{\boldsymbol{\tau}}\boldsymbol{\Phi}_{\boldsymbol{d}}\left( \boldsymbol{Q,\tau,D} \right)\boldsymbol{\#(}\boldsymbol{SEQ Equation \backslash* ARABIC}\mathbf{15}\boldsymbol{).} \end{aligned}$$

The result of the fit using the expression above is given in Supplementary Table 5.

|  | 3D | 2D | 1D |
| --- | --- | --- | --- |
| *R*_wp_ ( %) | 6.6 | 3.3 | 6.0 |
| *I*_100_, *I*_10_, *I*_1_ | 0.066(6) | 2.32(8) | 1.33(5) |
| *I*_011_, *I*_11_ | 0.088(8) | 1.0(1) |  |
| *I*_111_ | 0.00(1) |  |  |
| *I*_200_, *I*_20_, *I*_2_ | 0.076(19) | 3.9(2) | 0.96(10) |
| *I*_210_, *I*_21_ | 0.09(4) | 0.6(3) |  |
| *I*_211_ | 0.00(3) |  |  |
| *I*_220_ | 0.06(4) | 0.9(6) |  |
| *I*_300_, *I*_30_, *I*_3_ | 0.00(5) | 0.84(99) | 1.1(3) |
| *I*_310_, *I*_31_ | 0.03(3) | 0.40(98) |  |
| *C* | -2.7(4) | -1.23(16) | -0.54(17) |
| *A* | 0.60(5) | 0.36(3) | 0.019(45) |
| 2π*a*^-1^*D*^-1^ | 4.5(4) | 8.9(4) | 20(3) |

**Supplementary Table 5: Parameters obtained by fitting the equal time structure factor of pharmacosiderite to the *d*-dimensional (*d* = 1, 2, and 3) short-ranged order model.**

1. Zero-ENERGY modes of spin waveS in THE coplanar q = 0, Γ_5_ structure

We discuss the instability of the **q** = 0 and Γ_5_ structures in terms of the zero-energy modes in the *J*–*J*’ model. The **q** = 0 and Γ_5_ structures are apparently stable, as shown in Supplementary Fig. 14a, which depicts antiferromagnetic configurations between two tetrahedral spin clusters. For example, any Γ_5_ order with a nonzero q value is unstable because the intercluster couplings become ferromagnetic, as shown in Supplementary Fig. 14b for neighbouring clusters related to the time-reversal operation. Interestingly, as illustrated in Supplementary Fig. 14c, an antiferromagnetic intercluster coupling similar to that in the **q** = 0, Γ_5_ structure is also achieved by introducing a different irreducible representation of Γ_4_.


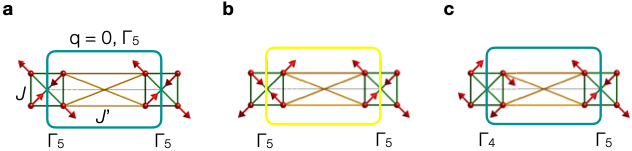


**Supplementary Fig. 14 | Comparison of the intercluster couplings.** **a**. **q** = 0, Γ_5_ magnetic structure viewed in the direction perpendicular to the coplanar plane. An antiferromagnetic spin configuration is realized in the elongated *J*’ tetrahedron. **b.** A pair of Γ_5_ type clusters related by the time-reversal operation expected for **q** = (1/2, 1/2, 1/2). A nearly ferromagnetic spin configuration is realized in the elongated *J*’ tetrahedron unlike **a**, which is energetically unfavorable. **c.** A pair of Γ_4_ and Γ_5_ type clusters. An antiferromagnetic spin configuration essentially the same as that in **a** is realized in the elongated *J*’ tetrahedron.

As discussed in the main text, the **q** = 0 and Γ_5_ structures possess a 2D character owing to dimensional reduction by frustration and are composed of decoupled layers perpendicular to the *c* axis (Supplementary Fig. 15). Here, we consider a 1D defect in the layer that may generate a zero-energy mode. When one Γ_4_ type cluster is introduced as a point defect into the **q** = 0, Γ_5_ layer, as depicted in Supplementary Fig. 15b, the neighbouring intercluster couplings become antiferromagnetic and ferromagnetic along the *a* and *b* axes with energies of – 2*J*’*S*^2^ and +2*J*’*S*^2^, respectively. Notably, the intercluster couplings along the *c* axis remain cancelled by frustration, even in this case. On the other hand, when a string of Γ_4_ clusters is created along the *b* axis instead of a single defect, as shown in Supplementary Fig. 15c, the energy-consuming ferromagnetic couplings along the *b* axis are removed and all the intercluster couplings become antiferromagnetic, so that the total energy remains the same as in the original Γ_5_ structure. This means that such 1D defects can easily occur in the layer of the Γ_5_ structure.

Remarkably, the spins in this 1D defect can continuously rotate without loss of energy. As illustrated in Supplementary Fig. 16, in each of the clusters of the 1D defect along the *b* axis, the Γ_4_- and Γ_5_-type configurations are transformed to each other by rotating the four spins cooperatively while keeping the total spin zero. There is no energy variation in the intercluster couplings to the surrounding clusters when this transformation takes place coherently in all the clusters of the 1D column. Thus, the defect can appear and disappear freely. The origin of this behaviour is the axial symmetry of the mean field from neighbouring clusters. The effective magnetic field exerted on the spins in the 1D defect from the pairs of spins in the next cluster points in the direction of *b* or –*b*, which allows a coherent “Larmor precession” around the *b* axis, as schematically depicted in Supplementary Fig. 16a. Specifically, the *ac* component of each spin in the column is rotated by an identical angle, and the *b* component is kept invariant. Therefore, a number of 1D defects are generated in the Γ_5_ structure as zero-energy modes, which may disturb the LRO and lead to a unique order with a large fluctuation. However, this may not be the case at *T* = 0 because small anisotropic interactions lift the zero-energy modes to gain a finite energy gap. Nevertheless, they are stable at elevated temperatures and may be frozen by crystalline defects at lower temperatures.

It is also noted that, owing to the tetragonal symmetry of the magnetic structure, similar 1D defects made of Γ_4_ type clusters can occur along the *a* axis. Then, one expects such a crossing of 1D defects, as depicted in Supplementary Fig. 16b. This is possible and does not increase the energy because one Γ_5_ type cluster is generated at the cross. This situation is in contrast to the case of the weathervane modes in the kagomé antiferromagnet, in which a crossing of two 1D defects is energetically unfavourable. This means that in pharmacosiderite, a number of 1D defects are generated along both the *a* and *b* axes and effectively weaken the LRO.


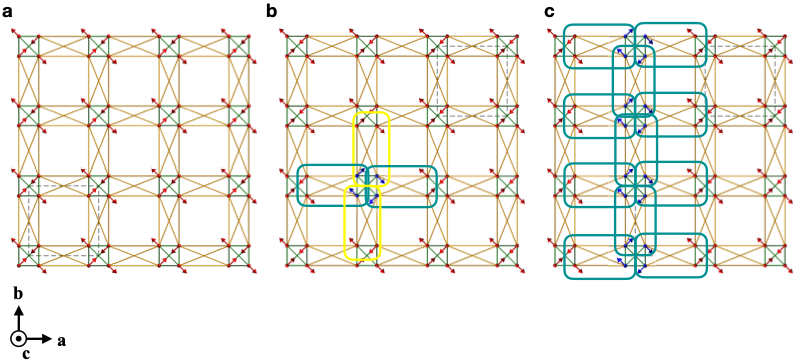
 **Supplementary Fig. 15 | 2D layer of the q = 0, Γ_5_ structure with Γ_4_ type defect clusters.** **a**. **q** = 0, Γ_5_ structure viewed along the *c* axis. Spins are aligned in the *c* plane. **b**. One of the clusters in **a** is replaced by a Γ_4_ type cluster with spins shown in blue. Antiferromagnetic inter-cluster couplings are realized along the *a* axis in the blue boxes, while nearly ferromagnetic couplings occur along the *b* axis in the yellow boxes, which increases the energy. **c.** 1D defect made of Γ_4_ type clusters along the *b* axis. The total energy is same as that in **a** because all the inter-cluster couplings become antiferromagnetic.


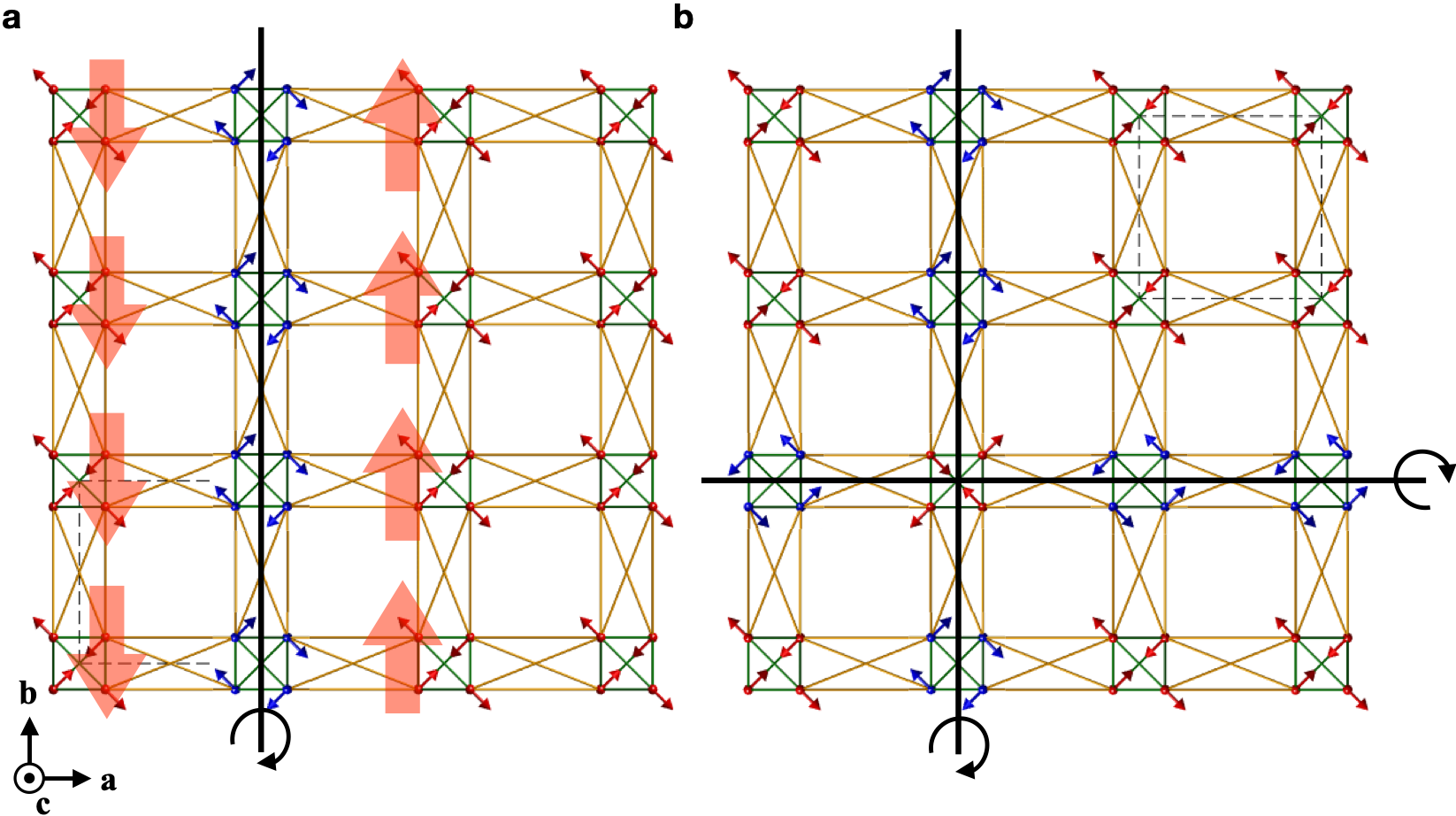


**Supplementary Fig. 16 | 1D zero-energy mode generated in the layer of the q = 0, Γ_5_ structure projected along the *c* axis.** The red and blue arrows represent spins having Γ_4_ and Γ_5_ arrangements, respectively. **a**. Axial symmetry in the column of Γ_4_ type clusters along the *b* axis. The large transparent arrow shows a total spin of two spins in a neighbouring cluster which generates an effective magnetic field along either *b* or –*b* on the spins in the defect column. This uniaxial nature allows continuous and coherent Larmor precession of all the spins in the defect column around the *b* axis without energy loss. Notably, a rotation by 180˚ leads to a transformation between the Γ_4_ and Γ_5_ arrangements. **b**. Intersection of two 1D defects. In addition to a 1D defect along the *b* axis, a 1D defect along the *a* axis can occur without any energy loss. At the crossing site, the Γ_5_ arrangement occurs, and all the intercluster couplings remain antiferromagnetic. Thus, a number of 1D defects can be generated in the layer at *T* = 0 in the absence of additional anisotropic interactions.

**Supplementary References**

1. Rodríguez-Carvajal, J. Recent advances in magnetic structure determination by neutron powder diffraction. *Physica B* **192**, 55 –69 (1993).
2. Okuma, R., Yajima, T., Fujii, T., Takano, M., & Hiroi, Z. Frustrated Magnetism of Pharmacosiderite Comprising Tetrahedral Clusters Arranged in the Primitive Cubic Lattice. *J. Phys. Soc. Jpn.* **87**, 093702 (2018).
3. Peterson, S.W. & Levy, H.A. A single-crystal neutron diffraction study of heavy ice. *Acta Cryst.* **10**, 70–76 (1957).
4. Blume, M. & Tjon, J. A. Mossbauer Spectra in a Fluctuating Environment. *Phys. Rev.* **165**, 446-456 (1968).
5. Rancourt, D. G., Julian, S. R., & Daniels, J. M. A new interpretation for the Mössbauer spectra of invar alloys; Anisotropic hyperfine field fluctuations. *J. Magn. Magn. Mater.* **51**, 83-88 (1985).
6. Brewer, J. H., Kreitzman, S.R., Noakes, D.R., Ansaldo, E.J., Harshman, D.R., & Keitel, R. Observation of muon-fluorine "hydrogen bonding" in ionic crystals. *Phys. Rev. B(R)* **33**, 7813–7816 (1986).
7. Jeffery, G.A. An Introduction to Hydrogen Bonding (Oxford University Press, New York, 1997).
8. Kadono, R., Shimomura, K., Satoh, K. H., Takeshita, S., Koda, A., Nishiyama, K., Akiba, E., Ayabe, R. M., Kuba, M., & Jensen, C. M. Hydrogen Bonding in Sodium Alanate: A Muon Spin Rotation Study. *Phys. Rev. Lett.* **100**, 026401 (2008).
9. Nishiyama K., Nishiyama S. W., & Higemoto W. Asymmetric F-μ-F interaction of the muon in polyfluorocarbons, *Physica B: Condens. Matter* **326**, 41-45 (2003).
10. Sugiyama J., Ikedo Y., Noritake T., Ofer O., Goko T., Månsson M., Miwa K., Ansaldo E. J., Brewer J. H., Chow K. H., & Towata, S. Microscopic indicator for thermodynamic stability of hydrogen storage materials provided by positive muon-spin rotation, *Phys. Rev. B* **81**, 092103 (2010).
11. Wills, A. S. A new protocol for the determination of magnetic structures using simulated annealing and representational analysis (SARAh). *Phys. B* (Amsterdam) **680**, 276–278 (2000).
12. Toth, S. & Lake, B. Linear spin wave theory for single-Q incommensurate magnetic structures. *J. Phys.: Condens. Matter* **27**, 166002 (2015).
13. Boysen, H. Analysis of diffuse scattering in neutron powder diagrams. Application to glassy carbon. *J. Appl. Cryst.* **18**, 320 –325 (1985).
